# The effect of NK cell therapy on sepsis secondary to lung cancer: A case report

**DOI:** 10.1515/biol-2022-0702

**Published:** 2023-08-31

**Authors:** Jingling Tang, Lulu Xie, Honglin Liu, Liyun Wu, Xiaoyang Li, Hang Du, Xinjun Wang, Xiaoyun Li, Yuan Yang

**Affiliations:** Clinical Research Center, Affiliated Hospital of Guizhou Medical University, Guiyang, Guizhou 550004, China; The Department of Critical Care Medicine, Affiliated Hospital of Guizhou Medical University, Guiyang, Guizhou 550004, China; Cancer Biotherapy Center, Affiliated Hospital of Guizhou Medical University, Guiyang, Guizhou 550004, China; Clinical Research Center, Affiliated Hospital of Guizhou Medical University, No. 28 Guiyi Road, Yunyan District, Guiyang, Guizhou 550004, China

**Keywords:** natural killer cell therapy, cytokines, sepsis, lung cancer, case report

## Abstract

Patients with sepsis face high mortality rates and a bleak prognosis, prompting the need for advanced therapeutic interventions. A male patient diagnosed with moderately low-differentiated squamous cell carcinoma received diverse treatments, including radiotherapy, chemotherapy, immunotherapy, and targeted therapy to inhibit angiogenesis. Subsequently, he developed sepsis after comprehensive treatment, and conventional antibiotic combinations proved ineffective in combating the infection. As an experimental approach, allogeneic natural killer (NK) cell infusion was administered. Following the NK cell infusion, the patient regained consciousness, and laboratory analyses showed reduced infection-related markers, suppressed serum inflammatory cytokines, and elevated anti-tumor cytokines. However, the therapeutic effect only lasted 2–3 days. *In vitro* investigations demonstrated that the allogeneic NK cell product reduced interleukin-6 levels in the patient’s serum. Moreover, subsequent co-cultivation of the NK cell product with the patient’s serum resulted in a decrease in the proportion of cytotoxic subpopulations of NK cells and a downregulation of the expression of NK-mediated killing molecules. In conclusion, adoptive transfusion of allogeneic NK cells may improve sepsis symptoms in patients with tumor-related sepsis. *In vitro* co-culture tests hold promise in providing predictive biomarkers for treatment effectiveness.

## Graphical abstract

**Figure:**
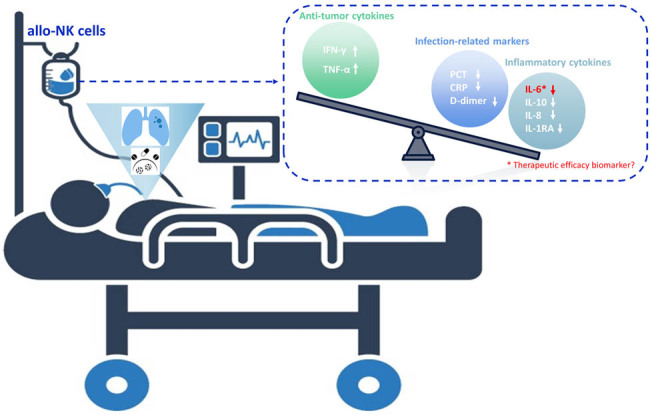

## 1 Introduction

Sepsis represents a critical condition characterized by organ dysfunction, bearing significant morbidity and mortality, arising from an imbalanced immune response by the host to infection [1]. Historically, a majority of research endeavors have focused on inhibiting tumor necrosis factor (TNF) and interleukin-1 to mitigate the inflammatory response [2].

During the course of sepsis, the immune system undergoes excessive activation, leading to heightened levels of circulating cytokines and hyperactivation of immune cells. Consequently, the immune response to the pathogen can contribute to the occurrence of multiorgan dysfunction and, in severe cases, even death [3]. Furthermore, even in instances where patients survive due to immunosuppression, long-term dysfunctional characteristics of immune cells persist, posing a life-threatening risk [4]. In recent years, it has come to light that natural killer (NK) cells hold promise as potential targets for therapeutic intervention, given their role as immune regulators. In this article, we present a case of sepsis secondary to lung cancer wherein the patient was treated with allogeneic NK cells.

## 2 Case report

A 57-year-old man presented with a persistent cough and hemoptysis persisting for a year and a half, without any prior history of chronic conditions such as diabetes, coronary heart disease, or chronic kidney disease. The patient had a long-standing history of hypertension and hyperuricemia, and the possibility of osteoporosis had been considered a year prior. In April 2019, he was diagnosed with intermediate-low differentiated squamous cell carcinoma of the left upper lung, with metastases observed in the liver, thyroid cartilage, and sternum at our medical facility. Genetic testing revealed negative *EGFR*, *KRAS*, *BRAF*, and *PIK3CA* mutations, while immunohistochemistry indicated a 5% expression of programmed cell death-ligand 1 (PD-L1) in the tumor tissue. Following multiple sessions of radiotherapy and anti-angiogenic targeted therapy, PD-L1 expression in the tumor tissue escalated to 63%.

In September 2020, the patient underwent a treatment regimen consisting of three doses of anti-tumor immunotherapy (durvalumab) in combination with chemotherapy (albumin paclitaxel) and targeted therapy (bevacizumab), accompanied by platelet-raising and leukocyte-raising measures.

During the course of the anti-tumor treatment, the patient experienced recurrent fever, immune system dysfunction, and unmanageable severe infection, culminating in sepsis and exacerbation of organ functionality impairment. Random blood glucose levels were higher than normal (Figure S1), although a diabetes diagnosis was not established due to the patient’s continued enteral nutrition. Three months later, the patient was admitted to the Intensive Care Unit (ICU) in a comatose state as a consequence of multiple comorbidities, including obstructive jaundice, multiple organ dysfunction syndrome, catheter-related bloodstream infection, intra-abdominal infection, concentrated toxic shock, lactic acidosis, fungal infection-induced necrosis of the thyroid cartilage, a postoperative abscess in the right pyriform fossa, subacute severe liver failure, hepatorenal syndrome, disseminated intravascular coagulation, toxicencephalopathy and hepatic encephalopathy, alimentary tract hemorrhage, severe anemia, and catheter-derived infection (Figure S2). Intermittent dialysis and plasma replacement therapy were initiated to address severe liver failure characterized by intermittent elevation of bilirubin levels, while red blood cell, plasma, and platelet transfusions were administered intermittently to address coagulation dysfunction. Etiological examinations suggested *Klebsiella pneumoniae* and *Enterococcus faecium* infections, complicated by refractory mucormycosis infection, which proved resistant to treatment involving multiple antibiotic regimens combined with anti-infective therapy (Tables S1 and S2). Despite treatment, the patient continued to experience recurrent fever, intermittent irritability, impaired consciousness, and progressive elevation of interleukin-6 (IL-6), C-reactive protein (CRP), and procalcitonin (PCT) levels.

Following treatment in ICU, the patient regained consciousness but remained in a critical condition. The effectiveness of anti-infection treatment was unsatisfactory, and the patient’s liver and kidney functions relied on supportive equipment for maintenance. In a multidisciplinary consultation (MDT), oncologists recommended against continuing standard anti-tumor therapy in this case and proposed considering allogeneic NK cell infusion for anti-tumor treatment and immune enhancement. After obtaining informed consent from the patient’s family, a total of seven infusions of NK cells were administered. The details of the NK cell infusion product were as follows: peripheral blood (100 mL) was collected from healthy donors without infectious pathogens. Peripheral blood mononuclear cells were isolated, and the cells were cultured *in vitro* using NK serum-free medium supplemented with cytokines IL2, IL15, and 5% autologous serum for a duration of 14 days. The release criteria for the product included a cell number of over 5 × 10^8^, cell viability after resuscitation of over 85%, and a proportion of NK cells (CD3-CD56^+^) of over 75%. The cells were suspended in saline containing 5% human serum albumin. The activation rate, indicated by the percentage of CD16-positive cells to all NK cells (CD3-CD56^+^ CD16^+^/CD3-CD56^+^), was found to be greater than 70% in both cases.

**Figure 1:**
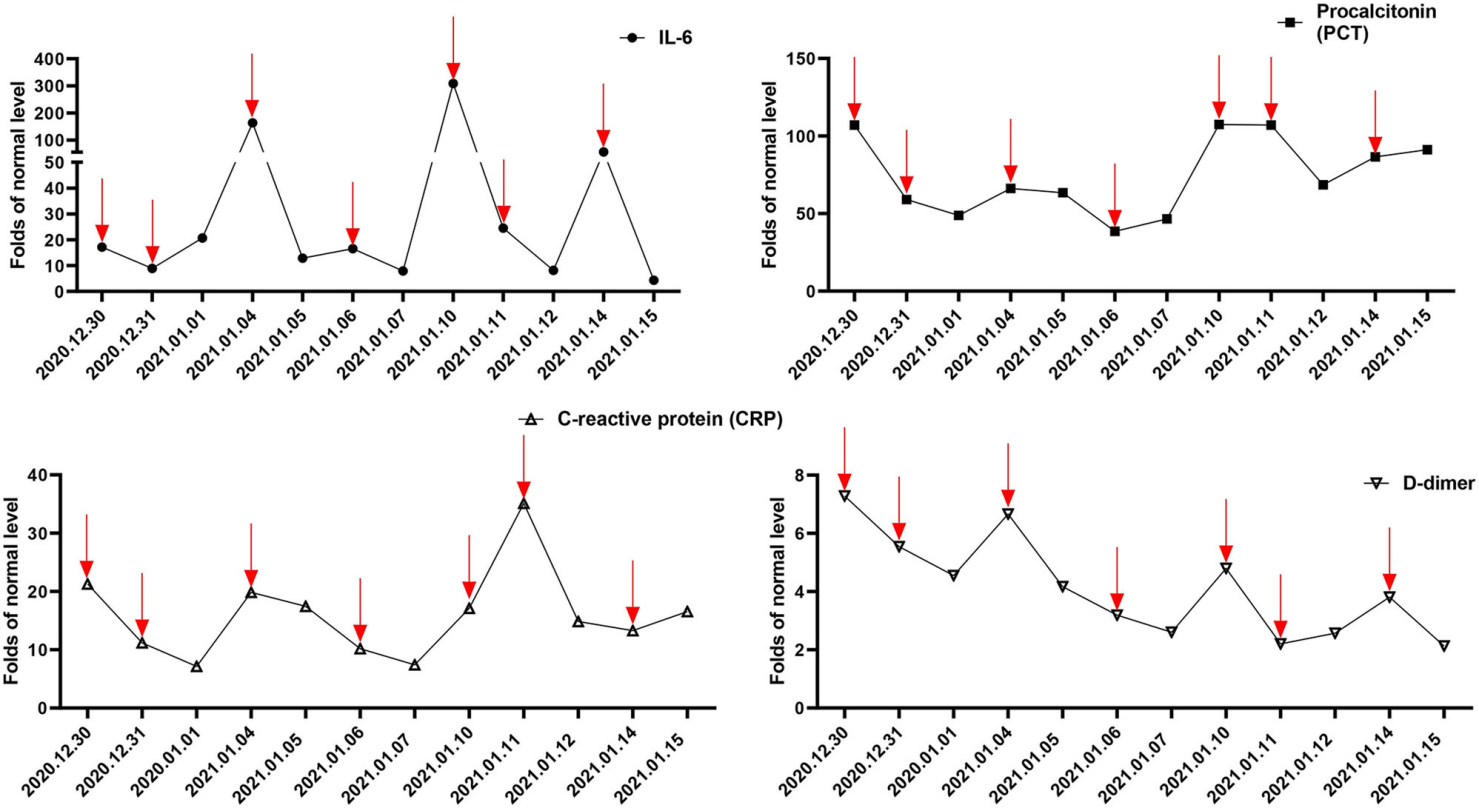
Change of serum sepsis biomarkers relative to normal level after NK cell infusion, including IL-6, PCT, CRP, D-dimer. Red arrows show the time of NK cells infusion. Red arrows indicate receipt of NK cell infusion on the same day.

**Figure 2:**
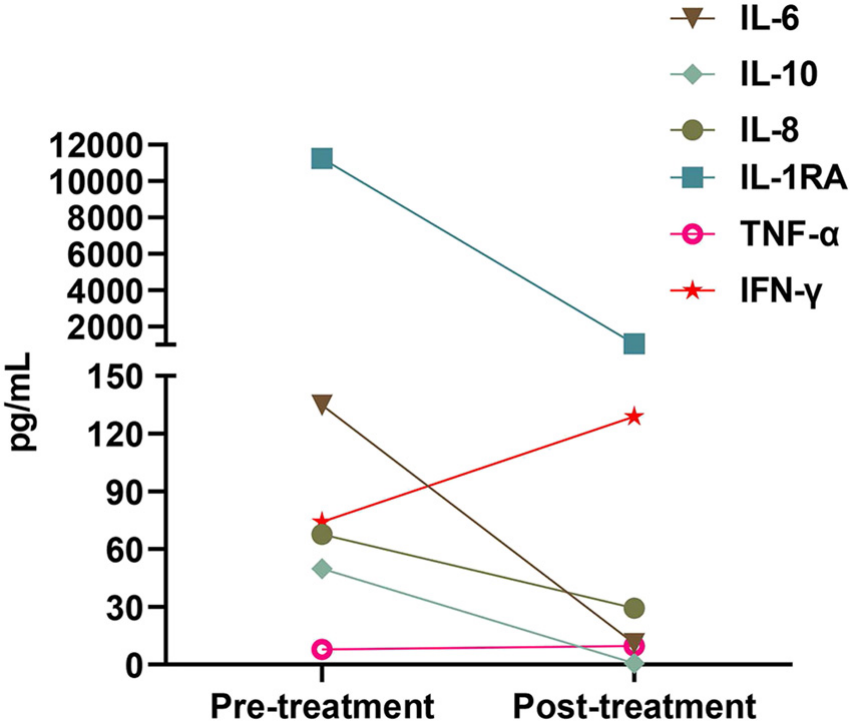
Alterations in serum levels of inflammatory cytokines (IL-6, IL-10, IL-8, and IL-1RA) and anti-tumor cytokines (IFN-γ and TNF-α) were assessed pre- and post-third NK cell infusion. A total of seven times of NK cells were infused, and the number of cells was 0.8–1 × 10^9^ each time. Serum samples were collected 8–12 h before NK cell infusion and 24–48 h after the last NK cell infusion.

After each cycle of NK cell infusion, the infection markers showed a decrease, and the patient experienced a recovery of consciousness and improved physical performance status. The laboratory data during the administration of NK cell infusions for seven cycles are depicted in Table S5. However, 2–3 days after the injection, the infection markers, including IL-6, CRP, D-dimer, and calcitonin, displayed an upward trend (Figure 1 and Table S3). Additionally, serum cytokine concentrations were measured before and after NK cell therapy. The data indicated that the treatment led to a decrease in serum levels of inflammatory cytokines (IL-6, IL-10, IL-8, and IL-1RA) and an increase in levels of anti-tumor cytokines (IFN-γ and TNF-α) (Figure 2 and Table S4).

Nevertheless, the infection indicators showed resurgence after 2–3 days post-injection. Considering immune hepatitis during a later MDT meeting, the patient was administered methylprednisolone at a daily dose of 40 mg on January 14, 2021, which was subsequently tapered. Following hormone treatment, the patient exhibited a progressive decrease in lymphocyte count (0.46 × 10^9^/L to 0.22 × 10^9^/L) and an increase in leukocyte count (9.2 × 10^9^/L to 13.19 × 10^9^/L). On January 21, the patient developed severe and uncorrectable acidosis, accompanied by a decrease in blood pressure, ultimately leading to demise.


**Informed consent:** Informed consent has been obtained from all individuals included in this study.
**Ethical approval:** The research related to human use has been complied with all the relevant national regulations, institutional policies and in accordance with the tenets of the Helsinki Declaration, and has been approved by Ethics Committee of Affiliated Hospital of Guizhou Medical University.

## 3 Discussion

### 3.1 Sepsis

Sepsis represents a syndrome characterized by the presence of multiple organ dysfunction, resulting from persistently elevated inflammation and immunosuppression [5]. The prevalence of sepsis is higher in individuals with cancer compared to those without, primarily due to the underlying malignancy or its treatment, which can heighten the risk of severe infections. Consequently, cancer patients are deemed to be at a heightened risk of experiencing elevated mortality rates from sepsis [6]. In the case at hand, the patient was in an advanced stage of lung cancer and had undergone radiotherapy, chemotherapy, and immunotherapy utilizing anti-PD-L1 monoclonal antibodies. The anti-tumor therapy inflicted damage upon functional organs, particularly radiation-induced liver injury, while immune system dysregulation and subsequent severe secondary infection further exacerbated organ dysfunction.

Throughout the progression of sepsis, the immune system becomes excessively activated, resulting in the production of excessive levels of cytokines [7]. Cytokines play a pivotal role in modulating the immune response by facilitating protective inflammation. However, when inflammation becomes excessive, it can lead to cellular damage, triggering further activation of the innate immune system and provoking a cascade of inflammation. This, in turn, can result in organ damage and dysfunction [3].

### 3.2 NK cell-based therapy

NK cells possess immunoregulatory properties that contribute to the maintenance of immune system equilibrium. Previous research indicates that allogeneic NK cells can attenuate graft-versus-host disease while simultaneously exerting a graft-versus-tumor effect [8].

NK cell-based therapies primarily target anti-tumor applications. These cells can directly eliminate tumors through various mechanisms, including cytokine secretion [5], induction of apoptosis [9], and antibody-dependent cell-mediated cytotoxicity [10]. Additionally, they can indirectly exert anti-tumor effects by modulating other immune cells such as T cells, B cells, and dendritic cells [11]. Current investigations in NK cell therapy encompass CAR-NK cell therapy [12] and non-genetically modified NK cell therapy derived from diverse sources, including peripheral blood, cord blood, and induced pluripotent stem cells [13].

While there have been reports of NK cell therapy being employed in the context of COVID-19 [14], there are currently no reports on the use of NK cell therapy for the treatment of severe sepsis. Previous studies have demonstrated that NK cells can be swiftly mobilized by danger signals and are among the earliest cells to reach target organs, including inflamed central nervous system tissues [15]. The role of NK cells in regulating inflammatory responses within different organs is often intricate and occasionally contradictory [16]. It has been observed that upregulation of NK cell activity can result in the downregulation of IL-1β and IL-6 expression [17]. In this particular case, short-term allogeneic NK cell therapy proved effective in normalizing the patient’s cytokine levels, potentially attributable to the reduction in IL-6 levels resulting from the co-cultivation of NK cells. Through *in vitro* experiments, we confirmed the capacity of allogeneic NK cells to deplete serum IL-6 in patients.

Regarding the cytotoxicity of NK cells, the infusion of unmodified NK cells, devoid of genetic modifications, exhibits a higher level of safety. This can be attributed to the fact that NK cell immune recognition is not restricted by major histocompatibility complex, enabling them to effectively target a broad spectrum of tumors while minimizing immune-related adverse effects [13]. In comparison to CAR-T cell therapy, the utilization of CAR-NK cells also leads to fewer severe side effects, such as cytokine storm and neurotoxicity, typically observed at grades 3–4 [12].

### 3.3 Case management

This study reports a case in which exploratory NK cell therapy was applied in a severe disease state, leading to temporary symptom relief. The study also explores the potential mechanisms underlying the improvement of symptoms in patients after NK cell therapy. In clinical practice, aggressive or exploratory advanced treatments are typically not employed in cases similar to this report. However, the treatment of this particular patient represents a breakthrough attempt. This case report suggests that NK cell therapy may be a worthwhile approach to consider for late-stage cancer patients with multiorgan failure and sepsis. Further exploration of the reproducibility of treatment effects is warranted, along with efforts to extend the duration of effective treatment through optimization of NK cells infusion and providing a window of time for the patient’s physical recovery. Additionally, the possibility of combining antibiotic therapy and/or anti-tumor treatment should be taken into consideration.

During the course of anti-infection treatment, despite continuous optimization of the antibiotic combination, the effectiveness of the treatment did not manifest prominently. Conversely, following treatment with allogeneic NK cells, the patient’s condition exhibited temporary improvement. Immune factors implicated in tumor development processes, such as IL-6, IL-10, IL-8, and IL-1RA, were suppressed, while anti-cancer immune factors, including IFN-γ and TNF-α, were activated in the patient’s peripheral blood. Furthermore, the levels of infection indicators (IL-6, PCT, CRP, and D-dimer) demonstrated a decline following each infusion. *In vitro* studies showcased the phenotypic characteristics of allogeneic NK cells, as illustrated in Figure S3a. The patient exhibited an increased proportion of peripheral NK cells subsequent to NK cell treatment (Figure S3b). Following overnight co-incubation of patient serum with allogeneic NK cells in 96-well plates, we assessed changes in IL-6 concentrations in the culture medium, observing that NK cells similarly reduced IL-6 levels in the serum *in vitro* (Figure S3c). The original therapeutic regimen remained unaltered during the NK cell infusion, thereby indicating a potential correlation between the therapeutic effect and the infusion of NK cells from a repeatability perspective.

Previous studies demonstrated that NK cells regulate alloreactive T cells and prevent the exacerbated immune responses [8,18]. Additionally, we observed a decrease in the absolute value of both helper and killer T cells (Figure S4), along with a tendency toward a decrease in lymphocyte levels (Figure S5) following NK cell treatment. However, as we did not analyze lymphocyte subsets, it is challenging to conclude that NK cells control sepsis through the regulation of T cell levels. Instead, we are inclined to believe that NK cells act as an “absorbent sponge” for inflammatory factors, based on the direct data of cytokine changes. Furthermore, we posit that the use of *in vitro* co-cultured NK cells with patient serum to detect changes in serum cytokines, as well as alterations in NK cell surface markers, may serve as potential biomarkers for assessing the effectiveness of NK cell therapy in the context of sepsis.

Following treatment with allogeneic NK cells, there was an observed increase in the proportion of activated subsets (cytotoxic subsets) of peripheral blood NK cells in the patient, along with an upregulation in the expression of activation molecules (NKG2D, DNAM-1, and NKP30/46) in peripheral blood NK cells (Figure S6a). Subsequently, we conducted tests to assess the changes in NK cells within the co-culture system using the same panel. Interestingly, the *in vitro* results contradicted the *in vivo* findings. The killing function of NK cells may have been impaired after the patient’s plasma treatment, as indicated by the reduction in the proportion of cytotoxic subpopulations and the downregulation of expression of NK-mediated killing molecules (Figure S6b), implying a depletion of NK cell function in the *in vitro* setting. It is plausible that the activated NK cells observed in the patient’s peripheral blood consisted of a mixture of NK cells derived from the patient himself and allogeneic NK cells, while the expression of functional molecules in allogeneic NK cells diminished during *in vitro* co-cultivation. We speculate that the infusion of allogeneic NK cells may have contributed to the enhancement of NK cell function in the patient himself. This may help explain the modest effect and limited duration of the NK cell therapy observed in the patient.

Regrettably, the NK cell treatment failed to achieve long-term efficacy. Following hormone therapy with methylprednisolone (40 mg qd, tapered), the patient’s peripheral blood immune cells continued to decrease, resulting in immune system collapse and secondary uncontrollable infection, ultimately leading to the patient’s demise due to uncorrectable acidosis. Pathogenic microbial infections have been shown to impair the function of immune cells, including NK cells and T cells, in patients, resulting in immunosuppression [19]. In fact, next generation sequencing testing identified multiple pathogen infections (Table S1), but we believed that this weakening effect takes a relatively long time to develop, and in this case, the loss of regulatory function within 48 h after allogeneic NK cells infusion was a rapid change. Therefore, we assume that the reasons for the short-term efficacy primarily lie in two aspects. First, the infused NK cells exhibited rapid apoptotic characteristics. During the *in vitro* culture of NK cells, high doses of IL-2 cytokines (500 IU/mL) were used for expansion, which may have caused the NK cells to become dependent on IL-2. Consequently, when removed from the IL-2 environment, NK cell activity and the expression of effector molecules were affected [20]. In other cases of NK cell-based therapies, subcutaneous injection of IL-2 is routinely performed as an adjuvant to sustain NK cell survival *in vivo*. However, in this particular case, the patient was critically ill with numerous complications, and we did not utilize IL-2 for maintenance due to safety considerations. This may be a contributing factor to the failure to sustain NK cell survival and activity following infusion into the patient’s body. Second, the duration of NK cell action was short-lived. From our perspective, the inability to maintain the efficacy of allogeneic NK cell infusion was linked to the rapid loss of NK cell numbers and function, which could potentially be improved by adjusting the treatment regimen. *In vitro* experiments demonstrated reduced NK cell activation, suggesting a similar phenomenon occurring after NK cells entered the patient’s body. This implies that NK cells may lose their function after interacting with inflammatory factors within the body. Therefore, the inability to sustain the efficacy of allogeneic NK cell infusion may be attributed to the rapid loss of NK cell numbers and function, which could be addressed through optimization of the treatment regimen.

The immune response of the host to sepsis undergoes a transition from an initial hyper-inflammatory phase to a prolonged immunosuppressive phase within a few days [21]. Although both proinflammatory and anti-inflammatory processes commence rapidly following the onset of sepsis, the initial hyperinflammatory phase typically prevails, potentially leading to shock, high fever, and multiorgan failure [22]. In patients experiencing sepsis as a secondary effect of oncology treatment, immune depletion accelerates tumor progression and hastens patient mortality [23]. Immunotherapy aimed at stimulating the immune system hold significant potential to reverse sepsis-induced immunosuppression and improve patient outcomes [24]. The modulation of the immune system to restore and reestablish adaptive immunity may emerge as a potent approach for future sepsis treatment [25]. In the present case, the infusion of NK cells is causally associated with the alleviation of sepsis symptoms and the reduction of inflammatory factors.

## 4 Conclusion

This case indicates that adoptive transfusion of allogeneic NK cells can alleviate symptoms in septic patients secondary to tumors. It may potentially serve as a therapeutic approach for septic patients, particularly those with sepsis related to tumors. However, on the other hand, addressing the issue of short duration of therapeutic efficacy in NK cell therapy and seeking improvement methods is also crucial.

## Supplementary Material

Supplementary material
