# Supplementary material for: The effect of NK cell therapy on sepsis secondary to lung cancer: A case report
Source: Open Life Sci. 2023 Aug 31;18(1):20220702. doi: 10.1515/biol-2022-0702 (PMC10476478; doi:10.1515/biol-2022-0702)
Supplement: Supplementary material [file biol-2022-0702-sm.pdf]

Supplementary material

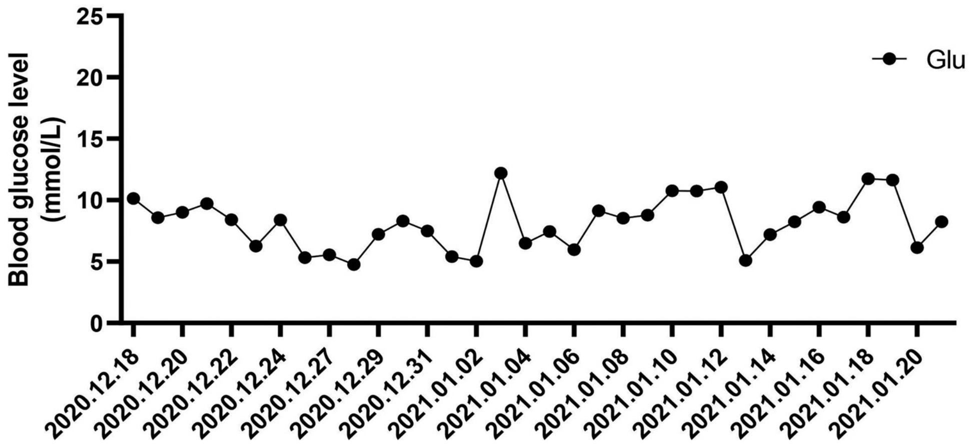

Figure S1: The patient's random blood glucose level during the treatment.

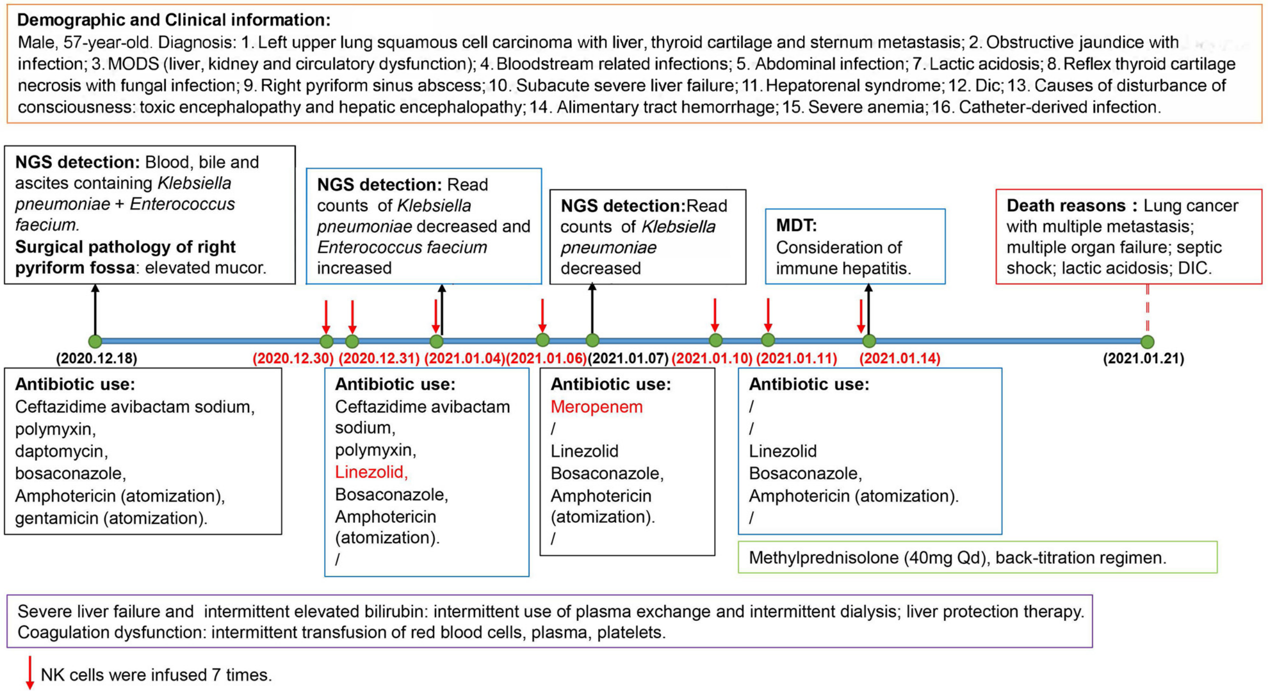

Figure S2: Demographic, clinical information, and treatment procedures.

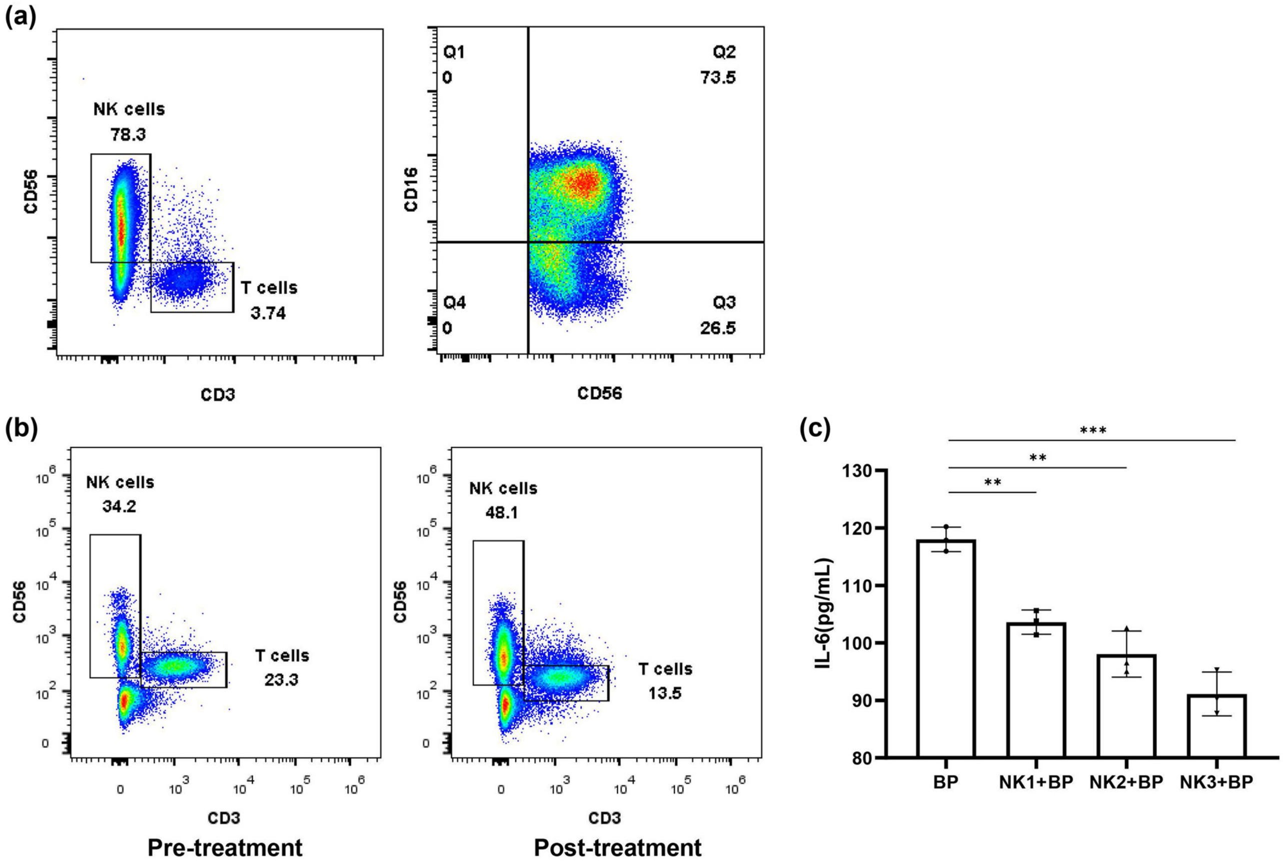

Figure S3: (a) Proportion of infused NK cells (CD3-CD56+) and activated NK cells (CD3-CD56+ CD16+/CD3-CD56+). (b) Elevated proportion of peripheral blood NK cells in patient after allogeneic NK cell treatment; (c) The effect of NK cells on serum IL-6 after co-culture overnight. The testing has been conducted on three different batches of NK cell products (NK1, NK2 and NK3). BP: Blood plasma.

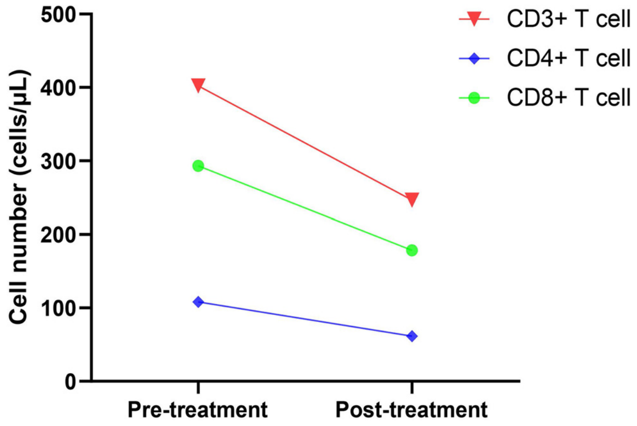

Figure S4: The absolute value of both helper and killer T cells before and after NK cell therapy.

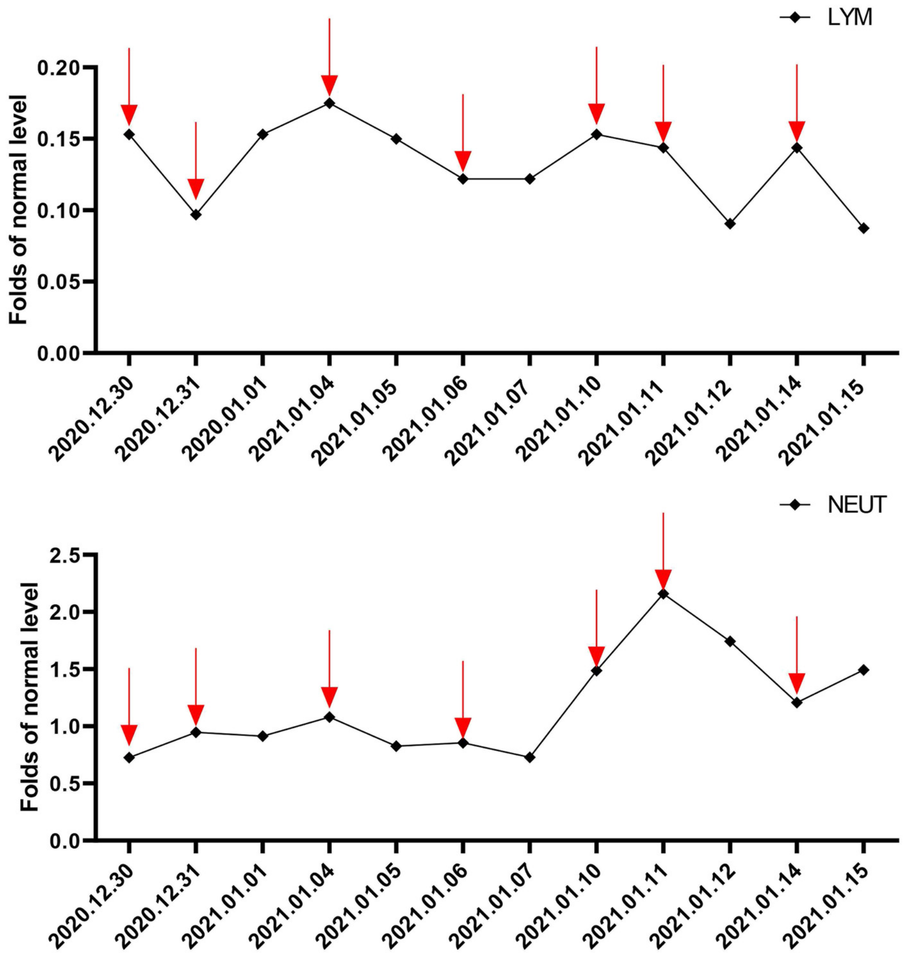

Figure S5: Change of lymphocytes and neutrophils level after NK cells infusion. Red arrows show the time of NK cells infusion.

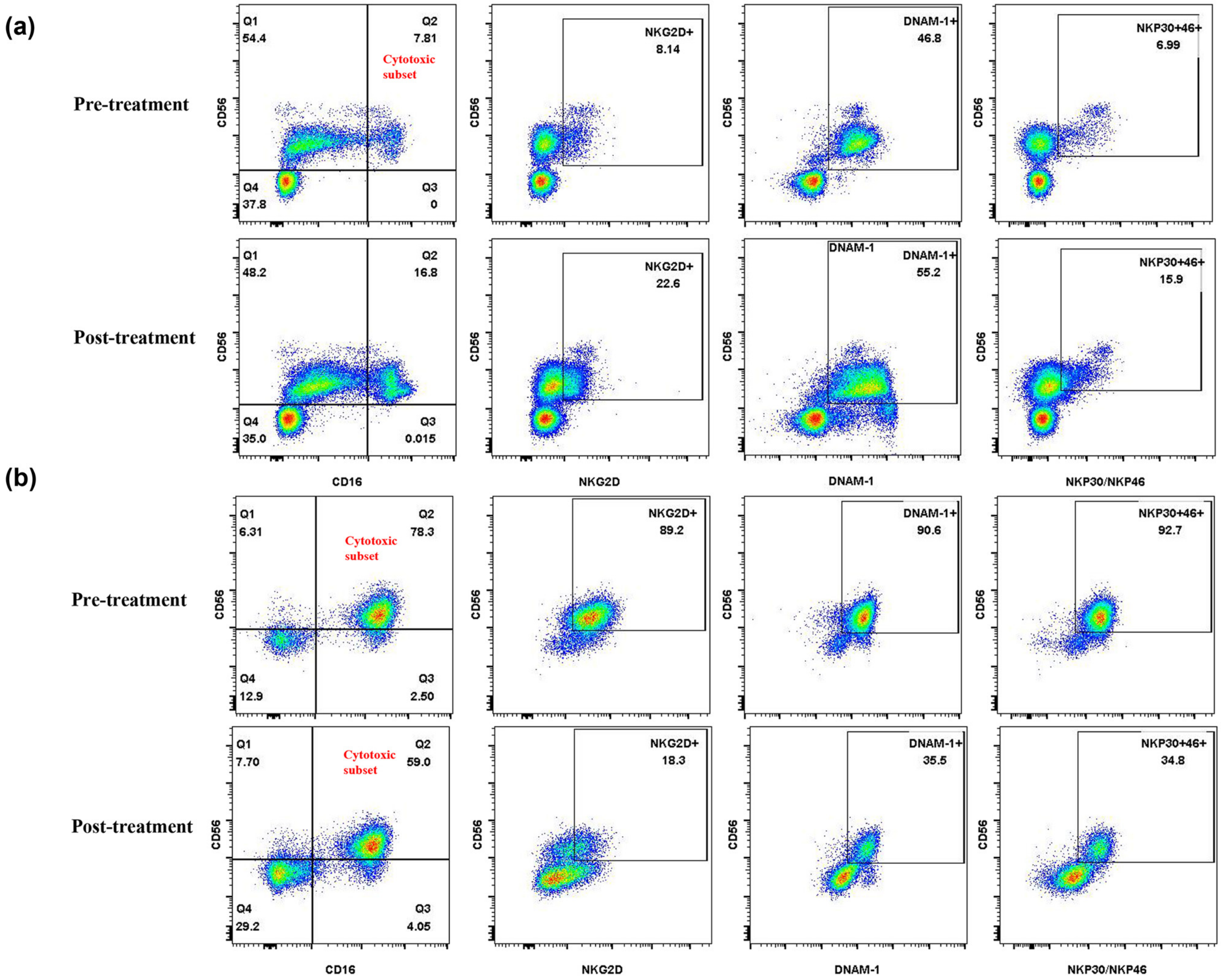

Figure S6: (a) After allogeneic NK cell treatment, the ratio of NK cell activation subset (Cytotoxic subset) and activation molecules (NKG2D, DNAM-1, NKP30/46) in peripheral blood of patient were increased. (b) Co-incubation of allogeneic NK cells with patient serum *in vitro* resulted in a decrease in the NK cell activation subset and a decrease in activation molecules.

Table S1: Patient's NGS test results

Date	Items	Sample	Microorganism types and sequence reads
2020.12.18	DNA + RNA	Bronchoalveolar lavage fluid	*Human α herpesvirus type 1*: 10
	DNA	Bile	*Klebsiella pneumoniae:* 370615
			Klebsiella heteroliticus: 9591
			Enterococcus faecium: 4198
			Enterococcus durans: 38
			Raoultella planticola: 1948
			Raoultella ornithinolytica: 192
			Candida tropicalis: 330
	DNA	Ascites	*Klebsiella pneumoniae*: 21
	DNA + Pro	Blood	*Klebsiella pneumoniae*: 5212
			Klebsiella heteroliticus: 134
			Human α herpesvirus type 1: 89
2021.01.04	DNA + RNA	Sputum	*Acinetobacter baumannii*: 15439
			Acinetobacter pittobacter: 121
			Enterococcus faecium:31
			Enterobacter hormaechei: 21
	DNA	Ascites	Negative
	DNA	Bile	*Klebsiella pneumoniae*: 51
			Enterococcus faecium: 50265
			Enterococcus durans: 245
			Weissella confuse: 3
	DNA + Pro	Blood	*Klebsiella pneumoniae*:150
			Klebsiella *: 4
			Human β herpesvirus type 5 (CMV): 34
2021.01.07	DNA + RNA	Sputum	*Acinetobacter baumannii*: 221269
			Acinetobacter pittobacter: 717
			Enterococcus faecium: 9019
			Enterococcus durans: 49
			Enterobacter hormaechei: 25
			Human β herpesvirus type 5 (CMV): 5
	DNA	Ascites	*Enterococcus faecium*: 1096
			Enterococcus durans: 4
	DNA	Bile	*Klebsiella pneumoniae*: 39
			Enterococcus faecium: 1054711
			Enterococcus hirae: 6909;
			Candida tropicalis: 2
	DNA + Pro	Blood	*Klebsiella pneumoniae*: 49
			Human β herpesvirus type 5 (CMV): 49
			Torque teno virus: 3
2021.01.16	DNA	Ascites	*Acinetobacter baumannii*: 16
			Human α herpesvirus type 1: 3

(Continued)

Table S1: *Continued*

Date	Items	Sample	Microorganism types and sequence reads
			Torque teno virus: 2
	DNA + RNA	Sputum	*Acinetobacter baumannii*: 198748
			Acinetobacter pittobacter: 1288
			Enterococcus faecium: 6
			Enterobacter hormaechei: 16
			Human α herpesvirus type 1: 277
			Human β herpesvirus type 5 (CMV): 30
	DNA	Bile	*Klebsiella pneumoniae*: 153
			Enterococcus faecium: 4779
			Enterococcus hirae: 37
			Weissella confuse: 3
			Candida tropicalis: 11

The detection results marked in red are the bases for antibiotic formulation; *Human β herpesvirus type 5* (*human cytomegalovirus*, CMV) detection results are marked in green.

Table S2: Antimicrobial susceptibility test results

Date	Sample	Microbial culture identification	Antimicrobial susceptibility test	
			**Sensitive antibiotic**	**Drug resistant antibiotic**
2020.12.29	Bile	*Enterococcus*	Ampicillin and salbactam	Ampicillin
				Ciprofloxacin
			Amoxicillin and Clavulanate	Erythromycin
			Potassium	Levofloxacin
			Ceftriaxone	Penicillin
			Clindamycin, Daptomycin	Rifampicin
				Streptomycin
			Furantoin	
			Gentamicin	
			Linezolid	
			Moxifloxacin, Trimethoprim and	
			sulfa	
			Tetracycline	
			Vancomycin	
2021.01.14	Bile	*Enterococcus faecium*	Ampicillin and salbactam	Ampicillin
				Ceftriaxone
			Amoxicillin and Clavulanate	Clindamycin
			Potassium	Ciprofloxacin
				Erythromycin
			Daptomycin	Levofloxacin
			Furantoin	Penicillin
			Gentamicin	Rifampicin
			Linezolid	Streptomycin, Trimethoprim and sulfa
			Moxifloxacin, Tetracycline	
			Vancomycin	
2021.01.24	Central line drainage fluid	*Acinetobacter baumannii*	Amikacin	Ampicillin and salbactam
			Ceftazidime and potassium	Ampicillin
			clavulanate	
			Cefoxitin	Aztreonam
			Cefazolin	Ceftriaxone
				Ceftazidime
				Cefotaxime
				Ciprofloxacin.

Table S3: Change of serum sepsis biomarkers relative to normal level before and after NK cell infusion

Biomarker	Detection value (fold of normal level)											
	First treatment		Second treatment	Third treatment		Fourth treatment		Fifth treatment		Sixth treatment	Seventh treatment	
	Pre-treatment	Post-treatment	Post-treatment	Pre-treatment	Post-treatment	Pre-treatment	Post-treatment	Pre-treatment	Post-treatment	Post-treatment	Pre-treatment	Post-treatment
	2020.12.30	2020.12.31	2021.01.01	2021.01.04	2021.01.05	2021.01.06	2021.01.07	2021.01.10	2021.01.11	2021.01.12	2021.01.14	2021.01.15
**IL-6 (pg/mL)**	120.00 (17.14)	62.19 (8.88)	144.60 (20.66)	1143.00 (163.29)	89.91 (12.84)	115.80 (16.54)	55.30 (7.90)	2163.00 (309.00)	171.60 (24.51)	56.93 (8.13)	390.30 (55.76)	30.28 (4.33)
**PCT (ng/mL)**	5.35 (107.00)	2.95 (59.00)	2.44 (48.80)	3.31 (66.20)	3.17 (63.40)	1.93 (38.60)	2.33 (46.60)	5.37 (107.40)	5.35 (107.00)	3.43 (68.60)	4.33 (86.60)	4.56 (91.20)
**D-dimer (μg/mL)**	7.28 (7.28)	5.54 (5.54)	4.55 (4.55)	6.66 (6.66)	4.17 (4.17)	3.19 (3.19)	2.60 (2.60)	4.80 (4.80)	2.20 (2.20)	2.57 (2.57)	3.81 (3.81)	2.13 (2.13)
**CRP (mg/L)**	106.41 (21.28)	55.85 (11.17)	35.67 (7.13)	99.19 (19.84)	87.22 (17.44)	50.85 (10.17)	37.12 (7.42)	85.50 (17.10)	175.53 (35.11)	74.33 (14.87)	66.41 (13.28)	82.77 (16.55)

IL-6, interleukin-6; PCT, procalcitonin; CRP, C-reactive protein. The fold of normal level in parentheses represents the ratio to the normal reference value. Normal serum reference values: IL-6: <7 pg/mL; PCT: <0.05 ng/mL; D-dimer: <1 μg/mL; CRP: <5 mg/L.

Table S4: Change of serum cytokines before and after NK cell infusion

Cytokine (pg/mL)	Before infusion (8–12 h)	After infusion (24–48 h)	Variation tendency
SDF-1α	225.7	556.6	↑
IL-1β	0.8	1.8	↑
IP-10	39.6	119.1	↑
IL-7	0.3	1.1	↑
Eotaxin	9.3	32.5	↑
IL-13	0.8	3	↑
IFN-γ	74.1	128.9	↑
TNF-a	7.9	9.6	↑
MIP-1 β	77.3	83.1	↑
MCP-1	15.3	29.1	↑
IL-18	159.4	204.6	↑
IL-6	134.8	10.9	↓
MIP-1α	2.4	8.4	↓
IL-8	67.7	29.3	↓
IL-10	49.8	0.7	↓
IL-1 RA	11255	1055	↓
RANTES	40.2	19.9	↓

Table S5: Laboratory data

	Reference Range	Minimum	Maximum	Mean ± SD
NEU (10^9/L)	1.8-6.3	4.57	14.83	8.63 ± 3.41
LYM (10^9/L)	1.1-3.2	0.25	0.56	0.40 ± 0.10
MONO (10^9/L)	0.1-0.6	0.32	1.26	0.75 ± 0.27
CRP (mg/L)	< 5	143.76	352.01	229.75 ± 62.42
Cr (μmol/L)	57-97	35.67	175.53	81.06 ± 35.60
ALT (U/L)	9-50	17.30	87.70	48.19 ± 15.90
AST (U/L)	15-40	51.30	165.30	86.30 ± 27.49
TBIL (μmol/L)	< 23	204.40	413.80	294.70 ± 56.50
DBIL (μmol/L)	≤8	129.50	267.90	182.00 ± 34.13
IBIL (μmol/L)	3-17	74.90	160.10	113.54 ± 24.03

NEU, Neutrophil; LYM, Lymphocyte; MONO, monocyte; CRP,C-reactive protein; Cr, creatinine; ALT, alamine aminotransferase; AST, aspartate aminotransferase, TBIL, total bilirubin; DBIL, direct bilirubin; IBIL, indirect bilirubin.
